# Analysis of Regional Differences in Asphalt Binder Under All-Weather Aging Based on Rheological and Chemical Properties

**DOI:** 10.3390/ma18122829

**Published:** 2025-06-16

**Authors:** Meng Guo, Yixiang Dong, Xu Yin, Mingyang Guan, Meichen Liang, Xudong Wang, Xiuli Du

**Affiliations:** 1 State Key Laboratory of Bridge Engineering Safety and Resilience, Beijing University of Technology, Beijing 100124, China; dyx589@emails.bjut.edu.cn (Y.D.); xuyin@emails.bjut.edu.cn (X.Y.); duxiuli@bjut.edu.cn (X.D.); 2 The Key Laboratory of Urban Security and Disaster Engineering of Ministry of Education, Beijing University of Technology, Beijing 100124, China; 3 Chongqing Municipal Facilities Operation Guarantee Center, Chongqing 400015, China; 19910133682@163.com; 4 School of Civil Engineering, Inner Mongolia University of Technology, Hohhot 010051, China; lmc@imut.edu.cn; 5 National Observation and Research Station of Corrosion of Road Materials and Engineering Safety in Dadushe Beijing, Beijing 101103, China; xd.wang@rioh.cn; 6 Research Institute of Highway Ministry of Transport, Beijing 100088, China

**Keywords:** asphalt binder, all-weather aging, climate-driven degradation, rheological and chemical properties, regional differences

## Abstract

Asphalt binder aging under natural exposure critically determines pavement durability, though current research inadequately captured performance evolution across diverse regional climates. This study investigated climate-driven degradation mechanisms through 12-month all-weather aging (AWA) tests in Gansu, Shandong, and Beijing via rheological (*G-R* parameter, stiffness modulus *S*-value) and chemical analyses (carbonyl index *I*_C=O_, sulfoxide index *I*_S=O_). The results demonstrated significant region-dependent aging disparities beyond laboratory simulation. In Gansu, extreme thermal fluctuations and UV radiation accelerated hardening via thermal stress cycles and photo-oxidation, yielding 52.4% higher *G-R* parameter than PAV. In Shandong, humid saline environments triggered sulfur oxidation-driven electrochemical corrosion, increasing *I*_S=O_ by 4.2% compared to PAV. In Beijing, synergistic UV–thermal oxidation elevated *I*_C=O_ and *S*-value by 8% and 40.7%, respectively versus PAV. Critically, *I*_C=O_ exhibited strong positive correlations with rheological degradation across regions (r > 0.90, *p* < 0.01). Based on *I*_C=O_, the 12-month all-weather aging rate in Beijing exceeded Gansu and Shandong by 18.5% and 68%, revealing UV–thermal coupling as the most severe degradation pattern. Novelty lies in quantifying region-specific multi-factor coupling effects (UV–thermal, hygrothermal–salt, etc.) and demonstrating their superior severity over PAV (Beijing > Gansu > Shandong). Dominant environmental factors showed distinct regional variations: UV radiation and temperature difference dominated in Gansu (*I*_C=O_, r = 0.76) and Beijing (0.74), while precipitation—*I*_C=O_ correlation prevailed in Shandong (0.76), yet multi-factor coupling ultimately governed aging. These findings provide theoretical foundations for region-tailored and climate-resilient asphalt pavement design.

## 1. Introduction

Asphalt pavements are susceptible to aging during long-term service due to various environmental factors such as high temperature, light, oxygen, and rain [1]. Aging leads to an increase in the hardness and brittleness of asphalt binder, significantly reducing the pavement performance and service life [2]. The behaviors of asphalt binder under natural exposure aging (NEA) critically determine pavement durability. The NEA method investigates asphalt binder performance evolution through authentic environmental conditions involving solar radiation, temperature–humidity cycles, and chemical interactions. NEA offers an advantage in data reliability, but it is limited by the long test period (typically ≥5 years). In the NEA practice, asphalt binder films are horizontally exposed to direct sunlight, or aged samples are obtained through in situ pavement sampling combined with extraction techniques. The NEA method provides a basis for studying field aging mechanisms of asphalt binder [3].

Most existing studies focus on isolated thermo-oxidative aging and ultraviolet (UV) aging. Zhao et al. compared environmental chamber, high-pressure pure oxygen condition, and the pressure aging vessel (PAV) test, revealing varying aging sensitivities of asphalt binder under different conditions. The thin film aging method simulating natural conditions (60 °C with aeration) demonstrated superior effectiveness, but UV and moisture factors were neglected [4]. Zhang developed a climate index model based on cumulative UV radiation flux (ΣUV_max_), showing a strong correlation (r = 0.91, *p* < 0.01) with rheological parameters of asphalt binder. It confirmed that climate-weighted indicators more accurately characterize aging progression than conventional time-based parameters [5]. Chen et al. found that 30 days of indoor UV aging were equivalent to 50 months of outdoor radiation based on the dual-reaction kinetics model [6]. Zhang et al. proposed equivalence relationships between laboratory and natural aging: 1-day UV aging ≈ RTFOT standard aging, 5 h PAV ≈ 1-year natural aging, and 20 h PAV ≈ 5-year natural aging [7].

Moisture effects and coupled thermal–oxygen–light–water interactions during asphalt binder aging were considered in some studies. Chen et al. established a kinetic model of humidity-thermal aging based on time–temperature equivalence principles, revealing that extreme temperatures (above 60 °C) accelerated the growth rate of softening point by 40% [8]. Wet–dry cyclic aging reduced carbonyl (C=O) and sulfoxide (S=O) group contents by 8.9–28.8% and 3.3–13.9%, respectively compared to thermal-oxidative aging, indicating the inhibitory effect of moisture on oxidation [9]. Li et al. improved the thermal–oxygen–water–light coupling aging method, showing that 5 h, 10 h, and 15 h of laboratory aging corresponded to 4, 6, and 7 years of NEA. It was significantly superior than the conventional thermal-oxidative aging method [10]. Liang et al. proposed a multi-factor accelerating (MFA) aging method integrating thermal–light–water effects. By establishing an aging prediction model based on the carbonyl index (*I*_C=O_) analysis of Beijing-exposed asphalt binders (3–5 years), they determined that 5 days of MFA aging were equivalent to 2 years of natural service, with 5% accuracy improvement over PAV [11]. These findings provide critical data support for establishing a standardized aging evaluation system.

The synergistic effect of intense UV and large temperature differences on asphalt binder had been investigated. Ran et al. conducted an NEA test on the Tibetan Plateau, revealing strong correlations (r > 0.9) between aging time, macroscopic properties and chemical functional groups (C=O and S=O), confirming the dominant role of functional groups in the deterioration of asphalt properties [12]. Bi et al. further found that NEA for 4–8 months was equivalent to laboratory accelerated aging (60 ± 2 °C with UV) for 2–3 days and 6 days. Based on this, correction coefficients (1.36–2.05) were introduced to refine the radiation equivalence model [13]. A comparative analysis between southern Tibet (with double the daily solar radiation of Chongqing) demonstrated 1.5–2 times faster aging rate in high-altitude regions compared to low-altitude areas [14].

Emerging researches had clarified distinct oxidation pathways between thermal-oxidative and photo-oxidative aging mechanisms. Wu et al. demonstrated that asphalt binder oxidation under high temperature, high pressure or continuous illumination followed oxygen adsorption-dominated mechanisms [15], while dehydrogenation prevailed under pure thermal conditions [16]. Specifically, thermal-oxidative aging primarily generated carboxyl groups through limited oxidation of aromatic alkyl side chains, accompanied by condensation–dehydrogenation reactions [17]. Thermal rupture of the C=C bond formed C=O, while the S–C bond oxidation produced S=O, increasing the oxygen-containing polar functional groups [18]. In contrast, photo-oxidative aging initiated free radical chain reactions via UV-induced C–H bond photolysis, exhibiting a carbonyl-to-sulfoxide formation ratio of 1.8:1 versus 1:2.1 in thermal conditions [19]. Although water exhibited weaker physicochemical impacts than UV radiation, it promoted the sensitivity of asphalt binder to photo-oxidation [20]. Yang et al. simulated practical service environments by immersing asphalt binders in distilled water, acid, alkali, NaCl, and Na_2_SO_4_ solutions [21]. It was shown that aqueous erosion increased the elastic components, stiffness, C=O and S=O, while reducing the fatigue life of asphalt binder [22]. Notably, acid, alkali, and salt solutes counteracted this trend [23].

The current research faces two primary limitations: (1) predominant focus on single environmental factor or laboratory accelerated aging, inadequately addressing synergistic degradation mechanisms under real-world multi-factor interactions; (2) insufficient systematic comparison of asphalt binder performance evolution across diverse regional aging conditions, thus failing to guide region-specific anti-aging designs. Therefore, Gansu (temperate, continental arid climate), Shandong (warm-temperate, semi-humid climate), and Beijing (warm-temperate, semi-humid monsoon climate) in China were selected as typical regions in this study. The climatic variations induced fundamentally differentiated aging mechanisms.

The objectives of this research are (1) to quantify spatiotemporal distribution characteristics of meteorological parameters (temperature, UV radiation, humidity, wind speed) in the Gansu, Shandong, and Beijing regions through the 12-month all-weather aging (AWA) test; (2) to analyze macro–micro performance evolution of asphalt binders via rheological (*G-R* parameter, stiffness modulus) and chemical indexes (*I*_C=O_, *I*_S=O_), with inter-indicator correlations; (3) to establish region-specific environmental coupling patterns through correlating climatic parameters with rheological and chemical properties degradation. This study provides theoretical support for material modification and optimization of pavement maintenance strategies.

## 2. Materials and Methods

### 2.1. Raw Materials

This study selected 90# virgin asphalt binder (Gansu Road and Bridge Shanjian Technology Co., Ltd., Lanzhou, China), with basic properties shown in Table 1.

### 2.2. Laboratory Accelerated Aging Tests

According to JTG E20–2011 [28], short-term and long-term asphalt binder aging were simulated using the Thin Film Oven Test (TFOT) and Pressure Aging Vessel (PAV), respectively. TFOT replicates thermal-oxidative aging during asphalt binder storage, transportation, and paving, which was conducted at 163 °C with a rotational speed of 5.5 r/min for 5 h. Subsequent PAV aging subjected the TFOT asphalt binder to 100 °C and 2.1 MPa pressure for 20 h.

### 2.3. Natural Exposure Aging Tests

#### 2.3.1. Climatic Characteristics of Different Regions

**Figure 1:**
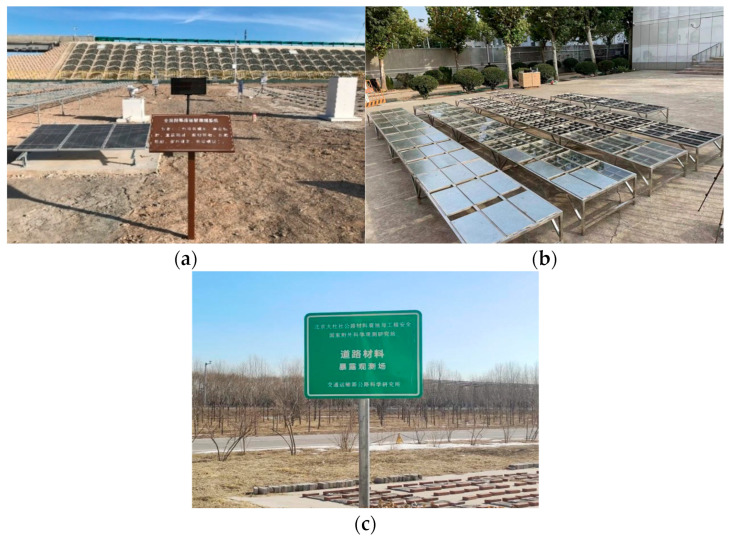
Field sites for all-weather aging tests in: (**a**) Gansu; (**b**) Shandong; (**c**) Beijing.

Field test bases in Beijing, Gansu, and Shandong were illustrated in Figure 1.

**Figure 2:**
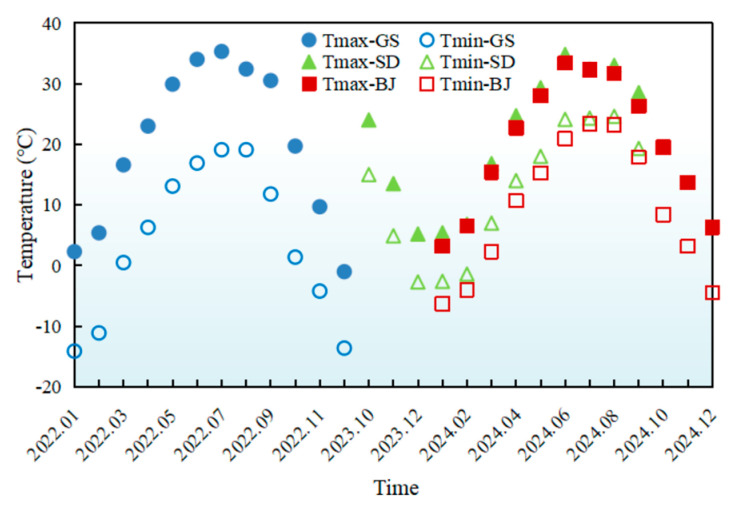
Monthly average variations in temperature of different regions.

**Figure 3:**
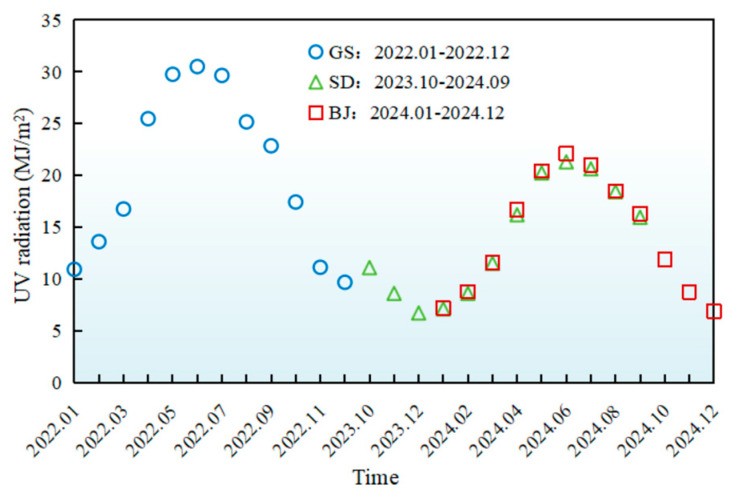
Monthly average variations in UV radiation of different regions.

**Figure 4:**
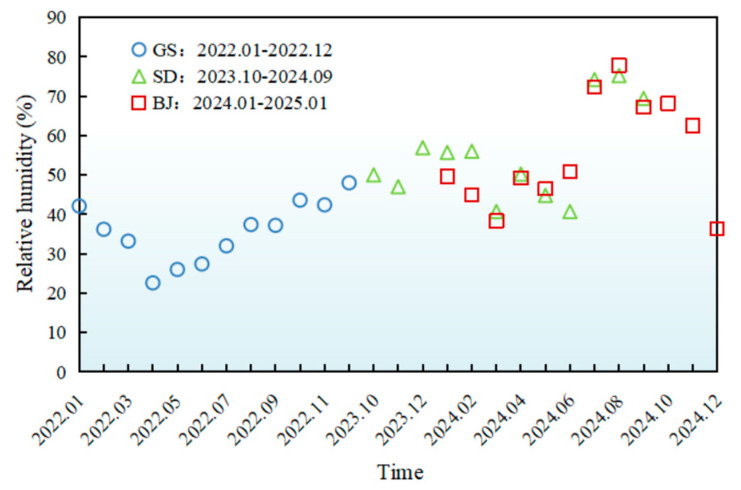
Monthly average variations in relative humidity of different regions.

**Figure 5:**
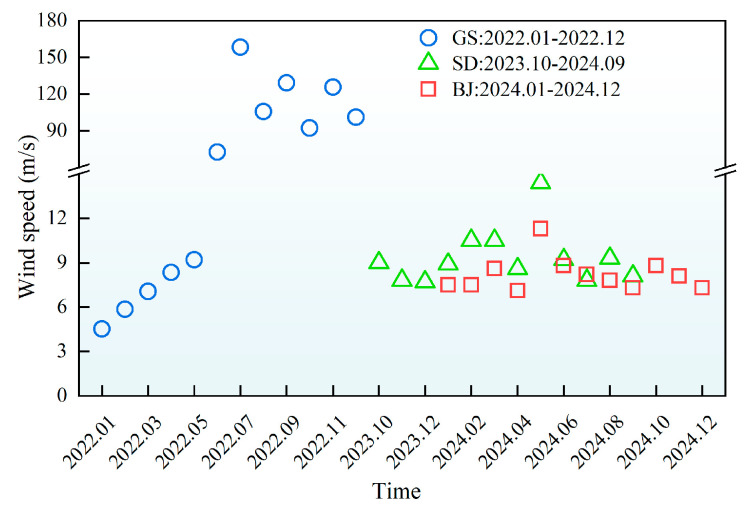
Monthly average variations in wind speed of different regions.

The monthly variations of meteorological parameters (temperature, UV radiation, humidity, wind speed) during AWA were presented in Figure 2, Figure 3, Figure 4 and Figure 5, with relevant abbreviations defined in Table 2.

In Figure 2, significant temperature variations were observed among the three regions. Gansu exhibited the most extreme temperature fluctuations with T_max_ 39.3 °C and T_min_ −19.6 °C, and monthly mean temperature difference of 18.7 °C. Such pronounced temperature extremes accelerated aging while challenging high and low temperature performance. In Shandong, moderated by maritime climate influences, temperatures stabilized between T_max_ 35 °C and T_min_ −3 °C, with a lower difference of 11.3 °C. Beijing displayed intermediate characteristics, recording T_max_ 33 °C and T_min_ −6 °C, and a temperature difference of 13.1 °C.

Figure 3 demonstrated similar trends between cumulative UV radiation and temperature variations across regions, peaking at 31 MJ/m^2^ (Gansu), 21.3 MJ/m^2^ (Shandong), and 22.5 MJ/m^2^ (Beijing). In Gansu, arid and cloudless conditions intensified ground-level UV exposure, significantly challenging UV aging resistance of asphalt pavement. In Shandong, seasonal UV fluctuations (20–22 MJ/m^2^ during May–Jul. vs. 6–9 MJ/m^2^ in Dec.–Feb.), reflected monsoon-driven cloud cover variations. In Beijing, UV levels consistently fell between Gansu and Shandong across all months.

Figure 4 and Figure 5 illustrated regional disparities in relative humidity and wind speed. In Gansu, an arid climate (annual average humidity 35.7%) combined with extreme winds (annual peak 158.4 m/s) intensified surface evaporation and dust activity. In Shandong, a maritime climate resulted in higher humidity (above 70% during Jul.–Sept.) and milder winds (9.3 m/s peak), reducing wind erosion risks. Beijing exhibited transitional climate characteristics, lower humidity (35–50%) during dusty months (Dec.–May) rising to 50–80% post-rainy seasons (June–Nov.), with urban structures mitigating wind impacts (8.5 m/s annual peak).

#### 2.3.2. Preparation and Processing of AWA Samples

**Figure 6:**
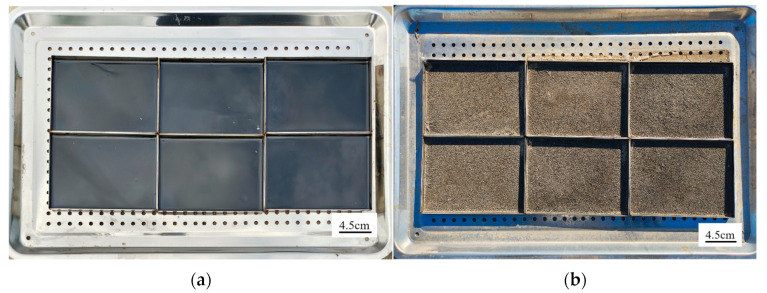
AWA test for asphalt binders: (**a**) before AWA; (**b**) after AWA.

The 90# virgin asphalt binder was heated at 135 °C until it flowed, with continuous stirring to avoid bubbles. Then, 30 g of asphalt binder was poured into 160 mm × 115 mm × 10 mm molds, achieving a film thickness of 1.59 mm (m = ρv). The samples were then subjected to TFOT. Next, the samples were placed at field test bases in Beijing, Gansu, and Shandong to undergo 12-month AWA without shield, exposed to sunlight, heat, oxygen, rain and dust, as shown in Figure 6. The samples were retrieved at 6-month intervals and processed as follows: surfaces were cleaned with damp cloths, air-dried at room temperature, heated to a flowable state at 135 °C, and stirred with a glass rod, and then cooled for performance testing. These steps were intended to minimize any interference from dust or other contaminants.

### 2.4. Characterization Methods of Rheological Properties

Dynamic shear rheometer (DSR, TA Instruments, New Castle, DE, USA) was used to evaluate intermediate-temperature fatigue resistance and low-temperature cracking resistance of asphalt binder.

(1) Intermediate-temperature fatigue resistance

Based on the high sensitivity of Glover-Rowe (*G-R*) parameter to aging, frequency sweeps (1% strain, 0.1–100 rad/s angular frequency) were conducted at 5 °C, 15 °C, and 25 °C with 8 mm parallel plates (2.0 mm gap) [29]. Master curves of complex modulus G* and phase angle *δ* were constructed at 15 °C, with values at 0.005 rad/s substituted into Equation (1) to calculate *G-R* parameters [30]. Increased G-R values indicate reduced fatigue resistance and elevated cracking risk [31].

(1) $$G-R=\frac{\left|G^{*}\right|\cdot {\left(\cos\delta \right)}^{2}}{\sin\delta }$$

(2) Low-temperature cracking resistance

While the *S*-value of 4 mm DSR correlates strongly with Bending Beam Rheometer (BBR) data [32], this study used the 4 mm DSR test (1% strain, 0.1–100 rad/s) at −18 °C, −12 °C, and −6 °C due to its sample efficiency advantage over BBR specimen preparation [33]. Increased S-value indicates impaired low-temperature cracking resistance and heightened failure risks [34].

### 2.5. Characterization Method of Chemical Composition

Spectrum II Fourier transform infrared spectroscopy (FTIR, PerkinElmer, Waltham, MA, USA) was employed in this study with the following parameters: 0.5 cm^−1^ resolution, 32 scans, and 4000–500 cm^−1^ wave. First, background spectra were collected prior to sample analysis. Molten asphalt was applied onto the testing platform for spectral acquisition. Then, OMNIC software (9.2) processed the spectra through transmission-to-absorbance conversion, followed by ordinate normalization, baseline correction, and smoothing. Absorption peaks of functional groups were identified with corresponding peak areas quantified.

## 3. Results and Discussion

The abbreviations and definitions of the samples were shown in Table 3.

### 3.1. The Effect of AWA on Rheological Properties of Asphalt Binders

#### 3.1.1. Evolution of Intermediate-Temperature Fatigue Resistance

**Figure 7:**
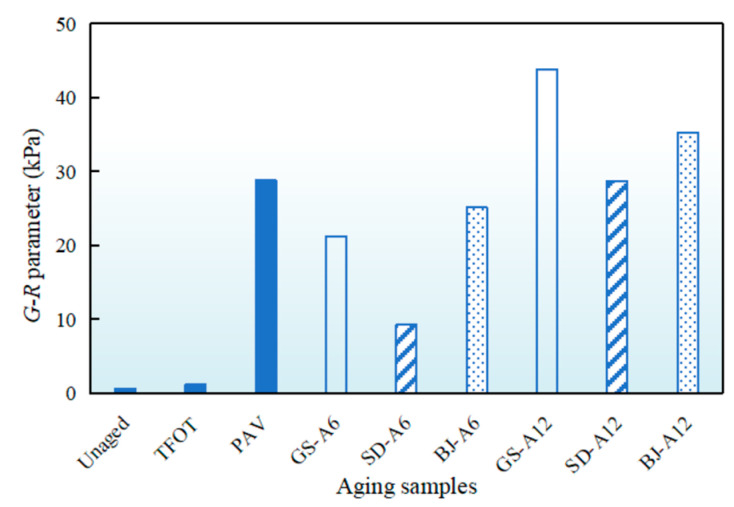
*G-R* parameters of asphalt binders after AWA in different regions.

*G-R* parameters of asphalt binders after AWA in three regions were shown in Figure 7. Unaged samples exhibited a *G-R* value of 0.6 kPa, and increased by 1 and 47 times after TFOT and PAV, respectively. Progressive *G-R* parameter growth occurred with increasing AWA time, showing regional disparities. After 6-month AWA, Gansu, Shandong and Beijing samples reached 74%, 32%, and 87% of PAV *G-R* values, respectively. After 12-month AWA, *G-R* parameters of Gansu and Beijing samples exceeded PAV 52.4% and 22.7%, respectively, while Shandong was 0.2% smaller than PAV. This direct comparison revealed that all-weather aging in Gansu and Beijing exceeded PAV severity, while in Shandong it was comparable. The results also demonstrated significantly greater fatigue resistance degradation in Gansu and Beijing compared to Shandong. In Gansu, cyclic effects of significant temperature fluctuations under all-weather conditions induced repeated expansion−contraction within asphalt binder, generating thermal stress and triggering microcrack initiation and propagation [35]. This degradation manifested as notable *G-R* parameter escalation. *G-R* parameters of all samples remained below the critical cracking value of 180 kPa, confirming compliance with specification requirements [36].

#### 3.1.2. Evolution of Low-Temperature Cracking Resistance

**Figure 8:**
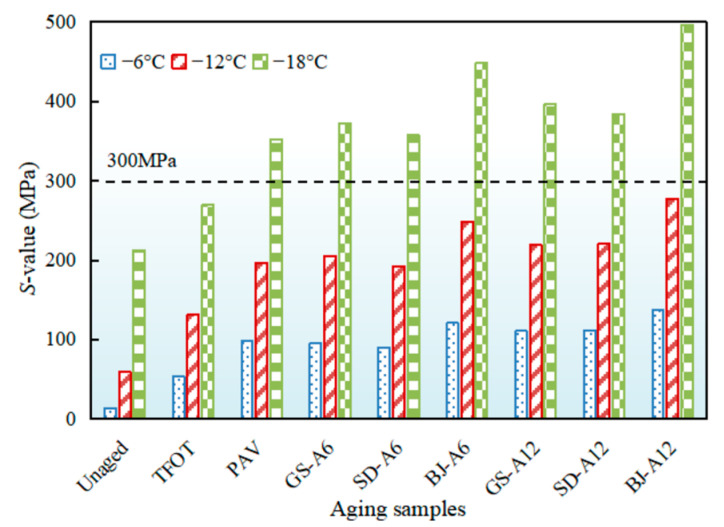
*S*-value of asphalt binders after AWA in different regions.

The *S*-value of asphalt binders after AWA in different regions was shown in Figure 8. As temperature decreased from −6 °C to −18 °C, the *S*-value exhibited significant increase with progressively amplified increments, indicating that the lower temperature, the worse cracking resistance of asphalt binders. An *S*-value at −18 °C exceeded 300 MPa except for unaged and TFOT, indicating failure to meet Superpave specifications for low-temperature deformation resistance [37].

**Figure 9:**
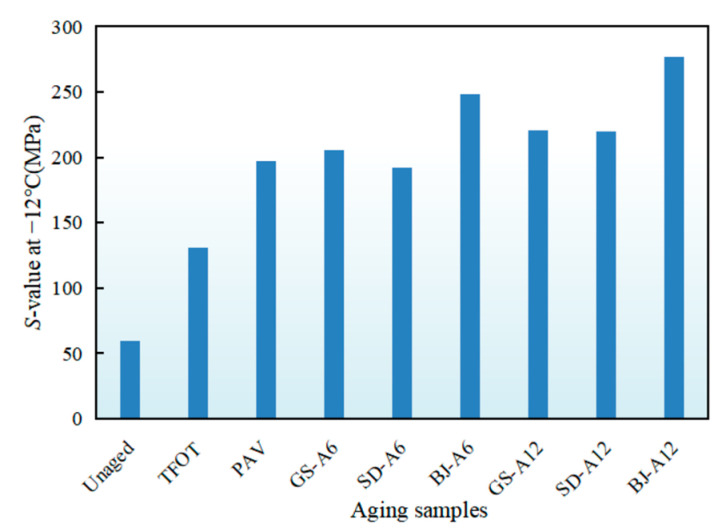
*S*-value at −12 °C of asphalt binders after AWA in different regions.

An *S*-value at −12 °C was selected for quantitative analysis, as shown in Figure 9. Unaged samples showed an *S*-value of 59.3 MPa, and increased by 1.2 and 2.3 times after TFOT and PAV, respectively. Beijing demonstrated the most pronounced aging effects; an *S*-value of BJ-A12 and BJ-A6 exceeded PAV by 40.7% and 26.1%. Gansu followed with GS-A12 and GS-A6 surpassing PAV by 12% and 4.4%, while Shandong exhibited minimal impacts with SD-A12 11.5% larger and SD-A6 2.6% smaller than PAV. Compared to PAV, all-weather aging induced significantly greater stiffness modulus increase in Beijing and Gansu, but was closer to PAV in Shandong. Critically, the comparison (*G-R* parameter, stiffness modulus) showed that all-weather aging in Beijing and Gansu often exceeded PAV severity in degrading rheological properties, highlighting the PAV potential underestimation in harsh climates.

The pronounced *S*-value increases in Beijing and Gansu were primarily attributed to thermal cycling-induced damage. Repeated temperature fluctuations generated internal stresses within the binder. As supported by Yu et al., these stresses could lead to the formation and propagation of microcracks in the asphalt binder microstructure [38]. These microcracks increased the binder’s surface area and facilitated oxygen ingress, thereby accelerating oxidative aging, which was a key mechanism for *S*-value elevation. The additional increase in Beijing was likely linked to acid rain exposure. Meng et al. demonstrated that acid rain components could interact with asphalt binder, leading to chemical changes such as the decomposition of lighter fractions (aromatics, resins) and a relative increase in asphaltenes [39]. This process disrupted the colloidal structure equilibrium, shifting it towards a more brittle gel state. Such structural degradation directly compromised the mechanical properties of asphalt binder, including increased stiffness (reflected in higher *S*-value) and reduced resistance to cracking, particularly at low temperatures.

### 3.2. The Effect of AWA on Chemical Functional Groups of Asphalt Binders

**Figure 10:**
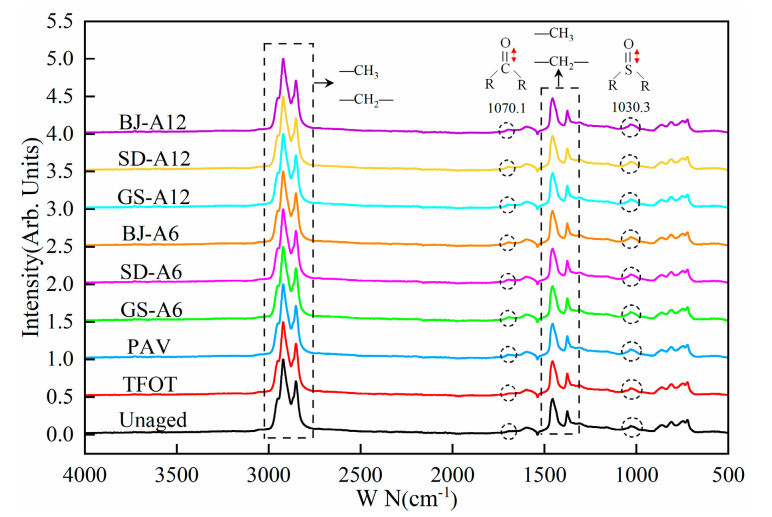
FTIR spectrogram of asphalt binders after AWA in different regions.

FTIR spectral characteristics of asphalt binders after AWA in different regions were shown in Figure 10. The characteristic peaks of methyl (-CH_3_) and methylene (-CH_2_-) groups (2852–2952 cm^−1^ and 1200–1550 cm^−1^) remained stable during aging, indicating minimal alkane structural changes. Therefore, this study selected the peak at 1459 cm^−1^ and 1378 cm^−1^ as reference peaks [3]. Notably, the carbonyl (C=O) peak near 1700 cm^−1^ emerged exclusively after PAV and AWA, confirming progressive oxidative accumulation. All samples exhibited persistent sulfoxide (S=O) peaks near 1030 cm^−1^, with peak area growth rates showing regional variability.

**Figure 11:**
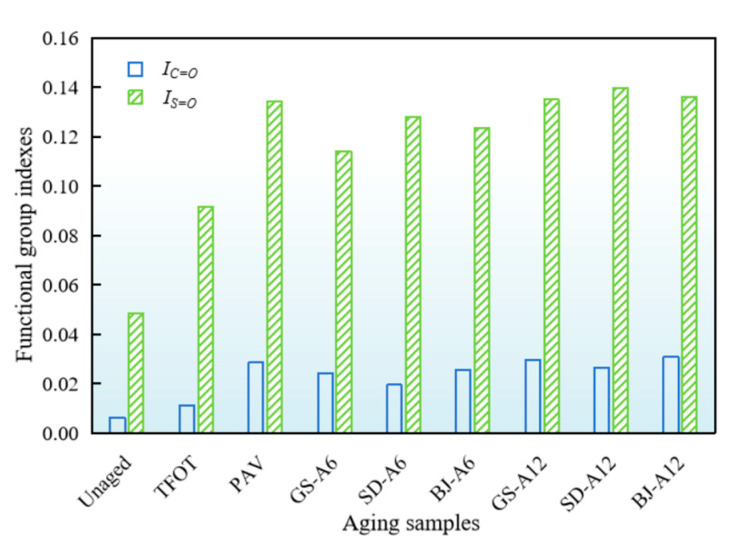
Functional group indexes of asphalt binders after AWA in different regions.

To quantitatively evaluate the aging degree of samples, the carbonyl index (*I*_C=O_) and sulfoxide index (*I*_S=O_) were employed to characterize the evolution of chemical composition, as calculated by Equations (2) and (3) and shown in Figure 11.

(2) $$I_{C=O}=\frac{A_{C=O}}{A_{ref}}$$

(3) $$I_{S=O}=\frac{A_{S=O}}{A_{ref}}$$

where *I*_C=O_, *I*_S=O_, *A*_C=O_, *A*_S=O_, *A*_ref_ represent the carbonyl index, sulfoxide index, carbonyl peak area, sulfoxide peak area, and reference peak area, respectively.

As shown in Figure 11, unaged samples exhibited *I*_C=O_ below 0.007, and increased to 1.7 and 4.4 times after TFOT and PAV, respectively. Both *I*_C=O_ and *I*_S=O_ showed an increasing trend with aging time, confirming that oxidation dominated functional groups accumulation during aging. High temperature, oxygen, and UV radiation collectively activated free radical generation, forming oxygenated polar functional groups such as C=O and S=O through reactions with C and S [40]. Obviously, the carbonyl sensitivities to different regions were as follows: BJ-A12 > GS-A12 > PAV > SD-A12 > BJ-A6 > GS-A6 > SD-A6 > TFOT > unaged samples. BJ-A12 exhibited the largest *I*_C=O_ (8% higher than PAV), followed by GS-A12 (3.8% higher than PAV), while SD-A6 showed minimal *I*_C=O_ (68.5% of PAV). *I*_C=O_ of other samples lied between TFOT and PAV. In Beijing, heavy metals (e.g., Fe, Cu) from atmospheric particulates adsorbed onto asphalt surfaces, catalyzing radical formation and indirectly elevating *I*_C=O_ [41]. In Gansu, low humidity reduced the antioxidative effect of moisture, intensifying molecular chain scission and oxygenated group accumulation [42].

However, *I*_S=O_ displayed different trends with *I*_C=O_. SD-A12, BJ-A12, and GS-A12 were 4.2%, 1.5%, and 0.7% higher than PAV, respectively. In Shandong, the coastal environment exposed asphalt binder to high levels of salt fog. Chloride ions (Cl^−^) derived from this salt fog readily combined with atmospheric moisture to form conductive electrolyte solutions on the asphalt binder surface. This facilitated electrochemical corrosion processes. Crucially, alkali metal ions (Na^+^) presented in the salt fog could act as catalysts, accelerating the oxidation of sulfur-containing compounds to form sulfoxides (S=O), thereby increasing *I*_S=O_. This catalytic effect and the role of the electrolyte in accelerating sulfur oxidation were strongly supported by the findings of Li et al. [43]. In Beijing, ${SO}_{4}^{2-}$ from acid rain could adsorb onto the asphalt surface and participate in reactions. Under acidic conditions, ${SO}_{4}^{2-}$ could promote the formation of surface complexes and facilitate radical chain reactions. These reactions specifically targeted sulfur-containing moieties within the asphalt binder, leading to their oxidation and the formation of sulfoxides (S=O), thus elevating the *I*_S=O_. The mechanism of ${SO}_{4}^{2-}$ in promoting oxidation under acidic conditions, particularly its pronounced impact on S=O formation compared to other aging factors, aligned with the observations reported by Qian et al. [44]. In contrast, the correspondence of *I*_C=O_ was more in line with a previous discussion on rheological properties [9].

### 3.3. Correlation Analysis of Macro and Micro Performance Indexes

**Figure 12:**
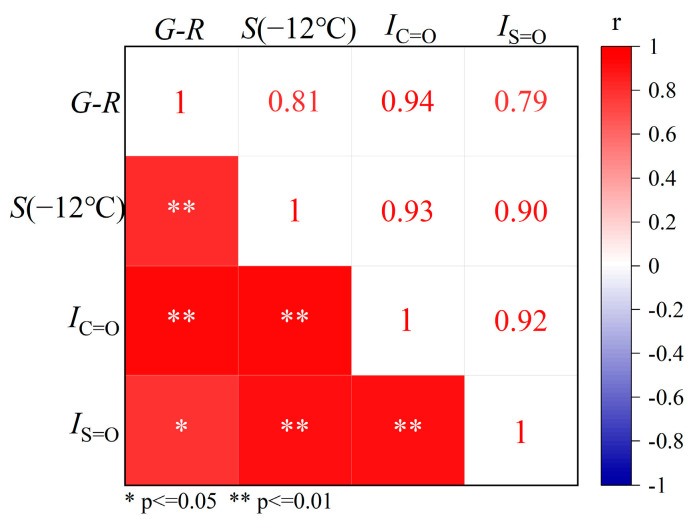
Pearson correlation results between indexes of asphalt binders.

Pearson correlation was analyzed between *G-R* parameters, *S*-value at −12°C, *I*_C=O_ and *I*_S=O_, with coefficients r calculated by Equation (4). Results were shown in Figure 12.

(4) $$r=\frac{\sum_{i=1}^{n}\left(X_{i}-\bar{X}\right)(y_{i}-\bar{y})}{\sqrt{\sum_{i=1}^{n}{\left(X_{i}-\bar{X}\right)}^{2}\sum_{i=1}^{n}{\left(y_{i}-\bar{y}\right)}^{2}}}$$

where Pearson correlation coefficient r ranges [−1, 1]. Correlation strength was classified as negligible (|r| < 0.3), weak (0.3 ≤ |r|< 0.5), moderate (0.5 ≤ |r|< 0.8), and strong (|r| ≥ 0.8).

As shown in Figure 12, the *G-R* parameter exhibited a strong linear correlation with *S*-value at −12 °C (r = 0.81, *p* < 0.01), indicating synergistic hardening behavior after all-weather aging [2]. Obviously, the results demonstrated that *I*_C=O_ exhibited the highest correlations with other indexes, with *G-R* parameter (r = 0.94, *p* < 0.01), *S*-value at −12 °C (r = 0.93, *p* < 0.01), and *I*_S=O_ (r = 0.92, *p* < 0.01). These strong and statistically significant correlations indicated that the accumulation of oxygenated polar group (as represented by *I*_C=O_) critically governed the rheological degradation of asphalt binder. Consequently, and in line with the findings of Lin et al. (2016) [45], the carbonyl area index (equivalent to *I*_C=O_) was verified as an effective parameter to characterize asphalt binder aging and was recommended as a critical chemical parameter for determining preventive maintenance time. *I*_C=O_ established itself as a robust chemical indicator for evaluating the aging-induced degradation relevant to medium-low temperature performance. The strong polarity of C=O in asphalt binder enhanced intermolecular forces, restricting molecular mobility and resulting in asphalt binder hardening with increased overall modulus. This structural alteration adversely affects rheological performance, compromising both fatigue resistance and low-temperature cracking resistance. Regionally, Beijing exhibited 18.5% and 68% faster AWA rates than Gansu and Shandong based on *I*_C=O_, respectively, indicating that all-weather environmental factors in Beijing led to the most serious aging, followed by Gansu, and Shandong was the smallest.

### 3.4. Correlation Analysis Between AWA Factors and Asphalt Binder Performance Across Regions

Previous findings revealed significant regional disparities in asphalt binder aging behaviors among Gansu, Shandong, and Beijing due to climatic variations. Quantifying environmental parameter impacts on performance metrics enabled the identification of region-specific critical aging factors. The abbreviations and definitions of environmental parameters were listed in Table 4.

**Figure 13:**
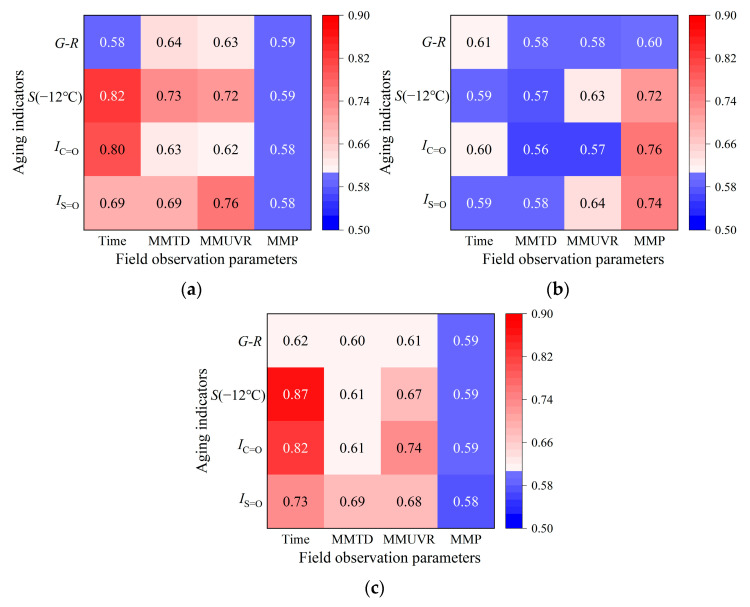
Correlation analysis results between field observation factors and aging indicators of asphalt binders after AWA in different regions: (**a**) Gansu; (**b**) Shandong; (**c**) Beijing.

Correlation matrices were constructed using aging duration, monthly temperature differentials, UV radiation, precipitation, and asphalt binder performance indicators (*G-R* parameters, *S*-value, *I*_C=O_, and *I*_S=O_). Figure 13 demonstrated distinct regional patterns.

As shown in Figure 13a, for AWA samples in Gansu, *S*-value and *I*_C=O_ exhibited the strongest correlations with aging time (r > 0.8), indicating that mechanical degradation and oxidation primarily depended on exposure time. Secondary correlations between *S*-value (r > 0.7), *I*_C=O_ (r > 0.6) and MMTD, and MMUVR further suggested synergistic degradation: thermal stress-induced microcrack and UV-driven molecular chain scission. Concurrently, *G-R* parameter correlations (r > 0.6) demonstrated combined thermal cycling (viscoelastic imbalance) and UV oxidation impacts. *I*_S=O_ strongly correlated with UV radiation (r = 0.76) and moderately with aging time and temperature difference (r = 0.69), indicating dual photochemical and thermo-oxidative sulfur oxidation. Degradation primarily stemmed from temporal accumulation accelerated by temperature fluctuations and UV, with negligible moisture influence (r < 0.3).

As shown in Figure 13b, AWA samples in Shandong exhibited strong correlations between MMP and *S*-value, *I*_C=O_, and *I*_S=O_ (r > 0.7), indicating moisture-driven degradation. Moderate UV radiation impacts (*S*-value, *I*_S=O_, r > 0.6) were attributed to humidity-induced UV scattering and slowed sulfide conversion. Weak correlations between aging time, temperature difference, and performance metrics (r ≤ 0.61) reflected the climatic stability of Shandong. Comparative analysis of Figure 2, Figure 3, Figure 4 and Figure 5 revealed limited thermal stress accumulation and reduced thermo-oxidative intensity compared to Gansu and Beijing, coupled with evenly distributed seasonal precipitation mitigating cumulative heat and drought effects. This stability diminished temporal and thermal degradation, positioning moisture-induced physicochemical deterioration, with secondary UV contributions, while climatic consistency significantly delayed thermo-oxidative progression.

As shown in Figure 13c, Beijing demonstrated the strongest correlations between aging time and all performance metrics (0.6 < r<0.9), followed by monthly UV radiation (0.6 < r<0.75) and temperature difference (0.6 ≤ r<0.7), with precipitation showing minimal influence (r < 0.6). These results indicated that asphalt binder degradation under all-weather conditions in Beijing was predominantly time-dependent, while UV radiation and thermal variations secondarily accelerated deterioration through physicochemical interactions. This pattern resembled Gansu’s aging dynamics but with weaker UV and temperature correlations.

Collectively, region-specific environmental coupling patterns governed asphalt binder aging. In Gansu’s arid climate, UV–thermal synergism (photo-oxidation and thermal stress) emerged as the primary pattern. Shandong’s moisture-rich environment accelerated hygrothermal–oxidation coupling with supplementary UV effects. Beijing exhibited integrated UV–thermal coupling effects that exacerbated degradation through combined photochemical and mechanical pathways. While each region displayed distinct primary patterns, the results conclusively demonstrated that asphalt binder aging in all regions resulted from coupled interactions of multiple climatic factors rather than a single factor.

## 4. Conclusions

This study conducted 12-month all-weather aging tests at field stations in Beijing, Gansu, and Shandong regions. Through spatiotemporal analysis of meteorological parameters and macro–micro evaluations of rheological properties (*G-R* parameters, stiffness modulus *S*-value) and chemical functional groups, region-specific aging patterns were revealed. The key findings are as follows:

1. All-weather aging patterns exhibited significant regional disparities. In Gansu, extreme temperature fluctuations and intense UV radiation caused 52.4% higher *G-R* parameter than PAV. In Shandong, hygrothermal–salt fog interactions promoted Cl^−^-induced electrochemical corrosion, leading to 4.2% higher *I*_S=O_ in SD-A12 than PAV. In Beijing, a synergistic effect of UV–thermal oxidation increased the *S*-value by 40.7% in BJ-A12 compared to PAV. Notably, all-weather aging in Beijing and Gansu induced more severe rheological degradation than PAV, while Shandong showed closer alignment, emphasizing region-dependent aging severity beyond laboratory simulation.
2. Chemical oxidation exhibited robust correlations with rheological properties degradation across all regions. *I*_C=O_ of BJ-A12 was highly linked with *G-R* parameter (r = 0.94) and *S*-value (r = 0.93), directly connecting oxygenated groups to hardening and performance degradation. Additionally, 49.5% faster C=O generation of GS-A12 than Shandong indicated that UV radiation prompted oxidation. Based on *I*_C=O_, the 12-month all-weather aging rate in Beijing surpassed Gansu and Shandong by 18.5% and 68%, respectively, demonstrating that UV–thermal oxidation coupling effects led to the most severe degradation pattern in Beijing.
3. Environmental parameters exhibited significant regional variations in impact intensity. Under all-weather conditions, UV radiation and temperature difference significantly accelerated aging in Gansu and Beijing (r > 0.6), with GS-A6 achieving 74% *G-R* parameter of PAV and BJ-A6 showing 26.1% higher *S*-value than PAV. The climatic stability of Shandong reduced thermal aging but displayed strong precipitation–carbonyl correlations (r > 0.7), with UV as a secondary factor. Yet crucially, multi-factor coupling effects resulted in aging rather than a single factor. Aging time impacted regions differently (Beijing > Gansu > Shandong).
4. It is noted that the indoor laboratory aging test (TFOT, PAV) in this study primarily simulated thermo-oxidative effects. Future research should incorporate multi-factor coupling (e.g., UV, moisture) to better simulate complex field environments. Future studies should extend aging time to capture long-term degradation, and develop region-specific anti-aging modifiers targeting dominant environmental stressors (e.g., UV stabilizers for Beijing and chloride inhibitors for Shandong).

## Figures and Tables

**Table 1:** Basic technical indexes of 90# virgin asphalt binder.

Properties	Results	Standards	Methods
Penetration (25 °C, 100 g, 5 s)/0.1 mm	81.8	80–100	ASTM D5 [24]
Softening point (R&B)/°C	46	≥44	ASTM D36 [25]
Ductility (5 cm/min, 10 °C)/cm	102	≥30	ASTM D113 [26]
Viscosity (60 °C)/Pa·s	144.5	≥140	ASTM D4402 [27]

**Table 2:** The abbreviations and definitions of meteorological parameters.

Abbreviation	Definition
T_max_-GS	Monthly average maximum temperature in Gansu
T_min_-GS	Monthly average minimum temperature in Gansu
T_max_-SD	Monthly average maximum temperature in Shandong
T_min_-SD	Monthly average minimum temperature in Shandong
T_max_-BJ	Monthly average maximum temperature in Beijing
T_min_-BJ	Monthly average minimum temperature in Beijing

**Table 3:** The abbreviations and definitions of the AWA samples.

Abbreviation	Definition
GS-A6	Samples after 6-month all-weather aging in Gansu
GS-A12	Samples after 12-month all-weather aging in Gansu
SD-A6	Samples after 6-month all-weather aging in Shandong
SD-A12	Samples after 12-month all-weather aging in Shandong
BJ-A6	Samples after 6-month all-weather aging in Beijing
BJ-A12	Samples after 12-month all-weather aging in Beijing

**Table 4:** The abbreviations and definitions of AWA observation factors.

Abbreviation	Definition
MMTD	Monthly mean temperature difference
MMUVR	Monthly mean ultraviolet radiation
MMP	Monthly mean precipitation

## Data Availability

The original contributions presented in this study are included in the article. Further inquiries can be directed to the corresponding author.

## Abbreviations

The following abbreviations are used in this manuscript:

AWA	All-weather aging
NEA	Natural exposure aging
MFA	Multi-factor accelerating
UV	Ultraviolet
RTFOT	Rolling thin film oven test
TFOT	Thin film oven test
GS	Gansu region
SD	Shandong region
BJ	Beijing region
GS-A12	Asphalt binders after 12-month all-weather aging in Gansu
DSR	Dynamic shear rheometer
BBR	Bending beam rheometer
FTIR	Fourier transform infrared spectroscopy
*G-R*	Glover-Rowe
*S*-value	Stiffness modulus
*I*_C=O_	Carbonyl index
*I*_S=O_	Sulfoxide index
MMTD	Monthly mean temperature difference
MMUVR	Monthly mean ultraviolet radiation
MMP	Monthly mean precipitation
